# Editorial: Immunosenescence and inflammaging: modulating immune function through diet, lifestyle, and therapeutic interventions

**DOI:** 10.3389/fimmu.2026.1897345

**Published:** 2026-06-18

**Authors:** Olivia Briceño, Iris P. Guzmán-Guzmán, Malén Massot-Cladera

**Affiliations:** 1 Infectious Diseases Research Center (CIENI), Instituto Nacional de Enfermedades Respiratorias Ismael Cosío Villegas, Mexico City, Mexico; 2 Universidad Autonoma de Guerrero, Chilpancingo, Mexico; 3 Physiology Section, Department of Biochemistry and Physiology, Faculty of Pharmacy and Food Science, University of Barcelona (UB), Barcelona, Spain; 4 Nutrition and Food Safety Research Institute (INSA-UB), University of Barcelona (UB), Santa Coloma de Gramenet, Spain

**Keywords:** immune function, immunosenescence, inflammaging, lifestyle, diet

## Abstract

**Figure:**
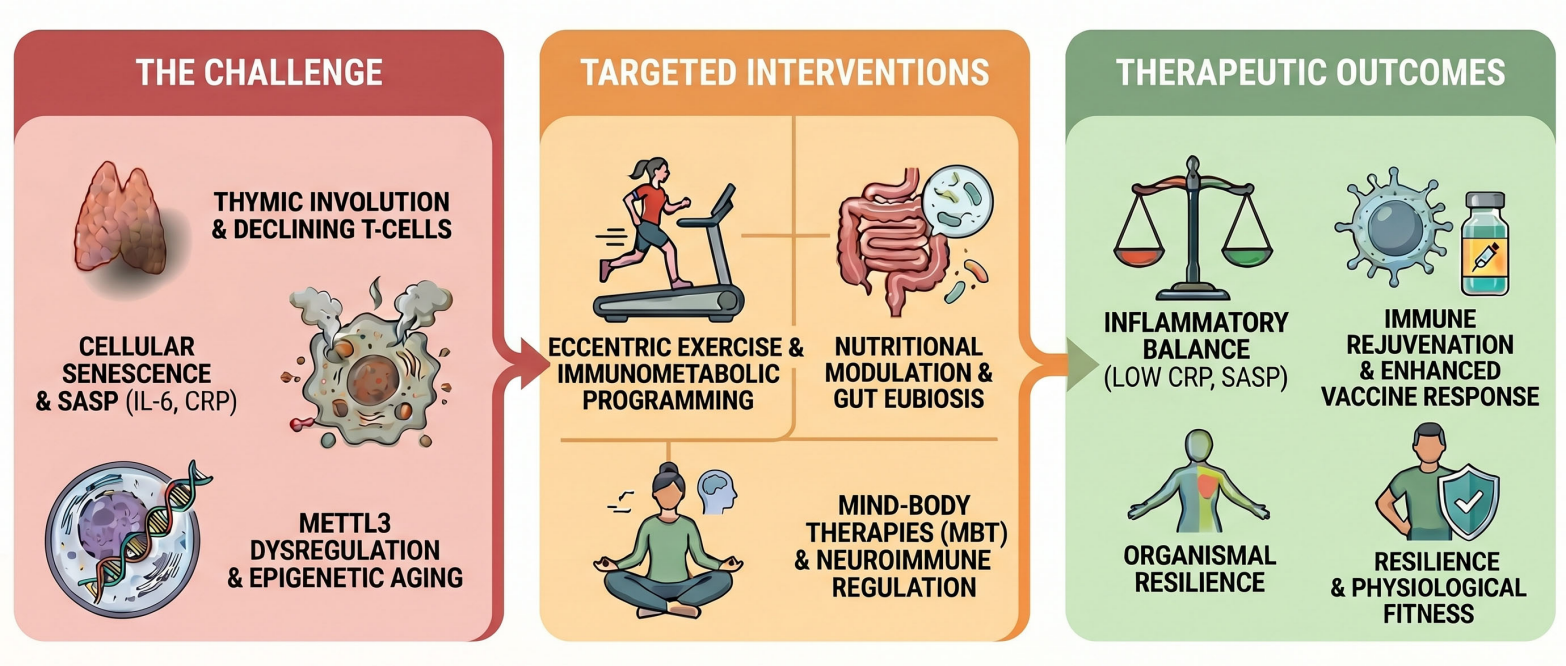

Aging is intricately linked to a progressive decline in immune system function, which increases the risk of cancer, autoimmunity, the development of metabolic syndrome and the susceptibility to acquire infections. This age-associated immune dysfunction, along with the dysregulation of the inflammatory processes, are respectively defined as immunosenescence and inflammaging.

The proper function and regulation of the immune system and the inflammatory responses are critical factors for organism homeostasis. While the health benefits of physical activity and immunonutrients supplementation have been documented, significant gaps remain in fully understanding their impact on health and disease states, particularly in elderly populations.

This Research topic includes a comprehensive review summarizing recent advances elucidating the mechanisms underlying immunosenescence and inflammaging. It also explores the strategies that have been tested to modulate aging, as integrative tactics that combine nutrition, microbiome modulation, and lifestyle interventions to sustain immune resilience across the lifespan (Müller and Di Benedetto).

Exercise has been explored as a potential modulator of immune function, though clinical evidence is still emerging. Within this Research Topic, original research demonstrates that moderate-intensity eccentric treadmill training improves skeletal muscle inflammatory cytokine profiles and restores insulin sensitivity (Luo et al.). Additionally, a systematic review analyzes the effects of physical activity on metabolic and inflammatory indicators in cancer patients, concluding that regular exercise confers modest, yet favorable effects modulating glucose, insulin and soluble factors of inflammation in this population (Wang et al.). Furthermore, another original study reveals that master athletes develop an adaptive immunoregulated profile that enhances cellular plasticity against inflammation, supporting that an active lifestyle is an effective non-pharmacological strategy capable of mitigating hyperreactive immune responses and effective for immune system regulation (Minuzzi et al.).

This Research Topic also includes a systematic review that synthesizes data from randomized controlled trials regarding the effectiveness of mind-body therapies on pro-inflammatory cytokine levels in patients with depression. The authors emphasize the need for further research to optimize therapeutic tools, adherence, and outcome measures in order to build a robust evidence-based framework for incorporating these therapies into nursing care (Mei et al.).

Understanding the molecular drivers of immunosenescence and inflammaging across diverse tissues and pathological conditions remains an urgent priority. In this Research Topic, original work about the molecular mechanisms influencing aging, particularly, epitranscriptomic modification and its regulatory interplay with microRNAs, is presented. Using senescence models in mouse skin fibroblasts, the authors demonstrated that silencing Methyltransferase-like 3 induces cellular senescence, whereas its overexpression delays senescent phenotypes by modulating mitophagy and skin aging. These findings suggest that pharmacological targeting of this molecular axis may hold therapeutic promise for age-related dermatological pathologies (Huang et al.).

In summary, this Research Topic offers a compendium of cutting-edge studies about mechanisms driving immunosenescence and inflammaging. It highlights promising non-pharmacological interventions such as physical activity and mind-body therapies, as well as molecular strategies aimed at delaying senescence. Collectively, these approaches attempt to modulate immune responses, improve metabolic health, and maintain long-term inflammatory homeostasis in aging and age-related pathologies.

